# Sequential Use of Apremilast, Total Glucosides of Paeony, and Periodontal Therapy in Refractory Plurimucosal Lichen Planus: A Case Report

**DOI:** 10.1155/crid/2405830

**Published:** 2025-11-20

**Authors:** Tinglan Yang, Zhenlai Zhu, Jiankang Yang, Mengmeng Song, Minghui Wei, Honglin Liu, Wen Qin, Shuai Shao, Chen Yu, Meng Fu, Qing Liu

**Affiliations:** ^1^State Key Laboratory of Oral & Maxillofacial Reconstruction and Regeneration, National Clinical Research Center for Oral Diseases, Xi'an, Shaanxi Province, China; ^2^Department of Oral Medicine, School of Stomatology, The Fourth Military Medical University, Xi'an, Shaanxi Province, China; ^3^Xijing 986 Hospital, Xi'an, The Fourth Military Medical University, Xi'an, Shaanxi Province, China; ^4^Department of Dermatology, The First Affiliated Hospital of Xi'an Jiaotong University, Xi'an, Shaanxi Province, China; ^5^Department of Dermatology, Xijing Hospital, The Fourth Military Medical University, Xi'an, Shaanxi, China

**Keywords:** apremilast, lichen planus, mucosal disease, PDE-4 inhibitor, total glucosides of paeony

## Abstract

Lichen planus (LP) is a chronic inflammatory disorder affecting mucosa and skin. This report presents a 61-year-old female with refractory LP involving buccal, gingival, and vulvovaginal mucosa, unresponsive to prior therapies including corticosteroids and hydroxychloroquine. The patient was treated with apremilast, a phosphodiesterase-4 inhibitor, combined with total glucosides of paeony. Within 4 weeks with apremilast and total glucosides of paeony, the patient experienced significant clinical improvement, with reduced Investigator Global Assessment and reticulation–erythema–ulceration scores. Periodontal scaling and root planing were performed at 8 weeks, further enhancing oral health. At 14 and 20 months after treatment initiation, the patient's condition remained stable, with no significant adverse effects. This case suggests that sequential therapy with apremilast and total glucosides of paeony may be a promising option for managing refractory LP involving multiple mucosal sites. Strategically timed periodontal intervention, initiated after systemic control, improved tolerability and underscored the value of a sequential, multidisciplinary approach.

## 1. Introduction

Lichen planus (LP) is a chronic inflammatory disorder that primarily affects the mucous membranes and skin, often proving resistant to topical treatments [1–3]. Lesions can occur on the skin, oral mucosa, nails, and genital areas, with oral manifestations typically presenting as white reticular erosive, or erythematous lesions. While cases with reticular lesions of oral LP are often asymptomatic, patients with erosive and erythematous lesions often present with pain or sensitivity, significantly impacting the quality of life [4]. Although the pathogenesis of LP is elusive, it is known to involve various inflammatory cytokines including interferon gamma (IFN-*γ*), interleukins (IL-2, IL-17, and IL-23), and tumor necrosis factor-alpha (TNF-*α*) [5, 6]. In addition to these immunological mechanisms, the etiology of LP has been associated with certain systemic diseases, most notably hepatitis C virus (HCV) infection [7, 8]. Furthermore, poor local oral hygiene is one of the contributing factors to the refractory oral LP. The accumulation of plaque and calculus resulting from inadequate oral hygiene management may further exacerbate the clinical symptoms of oral LP and increase the risk of periodontal disease [9–12].

Modern management of LP is guided by disease severity and site of involvement. Topical corticosteroids remain the first-line treatment for localized disease, while systemic therapies, including corticosteroids, retinoids, and immunomodulators, are often required for moderate-to-severe cases. For refractory cases who showed ineffective conventional therapies or multisite involvement, combination therapy would be used. In recent years, various small molecule drugs have been applied in the treatment of LP, including Janus kinase inhibitors such as tofacitinib, phosphodiesterase-4 (PDE-4) inhibitors such as apremilast, and biologics targeting the interleukin-23/interleukin-17 pathway [2]. Apremilast is an oral, small-molecule PDE-4 inhibitor that raises intracellular cyclic adenosine monophosphate (cAMP), which in turn modulates several immune responses, notably by reducing the production of the aforementioned cytokines [13]. Although Food and Drug Administration (FDA)-approved only for psoriasis and psoriatic arthritis, several studies have demonstrated the efficacy of apremilast in the treatment of LP [14–18]. Adverse effects (AEs) of apremilast include diarrhea, nausea, headache, and tension headache, among others [19]. These reactions typically emerge within the first 2–4 weeks of therapy, are mild to moderate in intensity, and resolve without intervention. Should symptomatic management prove insufficient and quality of life deteriorate, prompt referral to gastroenterology or neurology services is warranted for further evaluation and targeted treatment [20].

Total glucosides of paeony (TGPs) are considered to be the main bioactive ingredient of Paeoniae Radix Alba, and paeoniflorin is the main component of TGP [21]. This botanical preparation exerts immunomodulatory and anti-inflammatory effects and, since 1998, has held regulatory approval from the State Food and Drug Administration of China for the management of rheumatoid arthritis [21]. Earlier investigations in autoimmune disorders have demonstrated that TGP inhibits the production of inflammatory cytokines in oral LP by suppressing the NF-kappaB signaling pathway [22] and improves the immunomodulatory capacity of mesenchymal stem cells (MSCs) in oral LP [23]. In contrast to other immunosuppressive drugs, TGP modulates immunity bidirectionally, while conferring analgesic, liver-protective, and anti-inflammatory properties [21]. TGP produces only moderate therapeutic effects that exhibit efficacy slowly and gradually in patients with autoimmune disorders [21]. Therefore, rapid symptom control comparable to that achieved with synthetic agents is not anticipated. Combining TGP with synthetic drugs may nevertheless yield synergistic benefits and thereby attenuate their adverse-event profile. Moreover, TGP usually acts as a combination therapy for oral LP in China and increases efficacy [1, 24, 25].

In this report, we present a case of refractory LP affecting plurimucosal sites, including buccal, gingival, and vulvovaginal areas. Currently, no evidence-based guideline exists for vulvovaginal–gingival LP, underscoring the urgent need for novel therapeutic strategies. The patient's condition showed rapid improvement following treatment with oral apremilast and TGP, providing an opportunity for subsequent periodontal treatment to eliminate local recurrence factors.

## 2. Case Presentation

### 2.1. Case History

**Figure 1:**
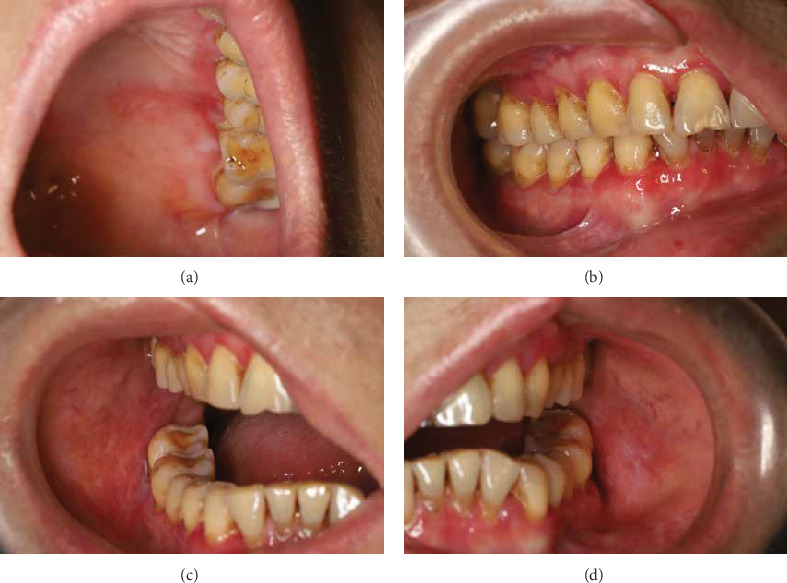
Clinical images of lichen planus involving left palatal mucosa (a), right gingival mucosa (b), right buccal mucosa (c), and left buccal mucosa (d).

A 61-year-old female presented with a 5-year history of recurrent white symmetric striations and erosion in the buccal and gingival (Figure 1) mucosa, which was aggravated in the last 1 month. Her medical history was significant for acute hepatitis C and pulmonary tuberculosis in her 20s. Her hepatitis C was resolved, with subsequent tests consistently showing negative results for hepatitis C RNA PCR over the past two decades. The pulmonary tuberculosis was cured after 1 year of treatment. She reported a history of glaucoma with well-controlled intraocular pressure in the past 3 years. She was treated primarily with corticosteroids or tacrolimus ointment, which resulted in the improvement of her oral lesions. However, the patient developed erythema and erosion in plurimucosal sites including vulvovaginal mucosa, which was resistant to oral hydroxychloroquine sulfate. Then, 1 month ago, she had a recurrence of the disease due to mental stress and flaring of both oral and vulvovaginal symptoms.

Physical examination revealed white striation and congestion in the posterior third of the buccal mucosa, where the left side was more prominent. The gingival erosion and redness were also observed, which were painful when brushing. Poor oral hygiene was observed with substantial plaque accumulation, dental calculus (++), and pigmentation (++). Bleeding on probing, gingival recession of 1–3 mm, 3–5 mm probing depths, and subgingival calculus were detected. The patient refused the vulvovaginal examination due to tenderness. Moreover, a full-body skin examination revealed the presence of longitudinal ridging on the fingernails. A diagnosis of LP was made based on the clinical symptoms.

### 2.2. Methods

After shared decision-making, she opted to be treated with apremilast combined with TGP. Treatment with apremilast was initiated at a dose of 10 mg on Day 1 and gradually increased over a week to the recommended dose of 30 mg twice a day.

### 2.3. Conclusions and Results

**Figure 2:**
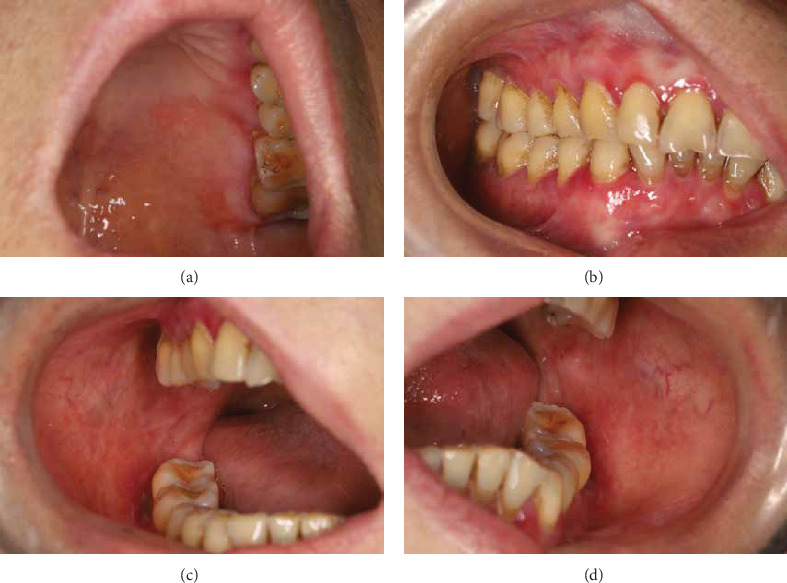
Clinical images at 4 weeks of left palatal mucosa (a), right maxillary and mandibular gingiva (b), right buccal mucosa (c), and left buccal mucosa (d).

**Figure 3:**
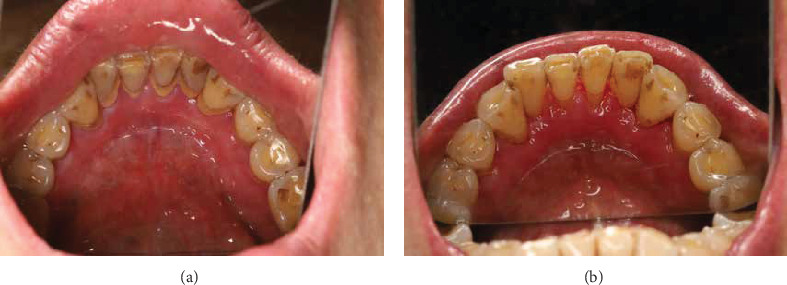
Clinical photographs of the lingual surfaces of the mandibular tooth before (a) and after (b) periodontal scaling.

**Figure 4:**
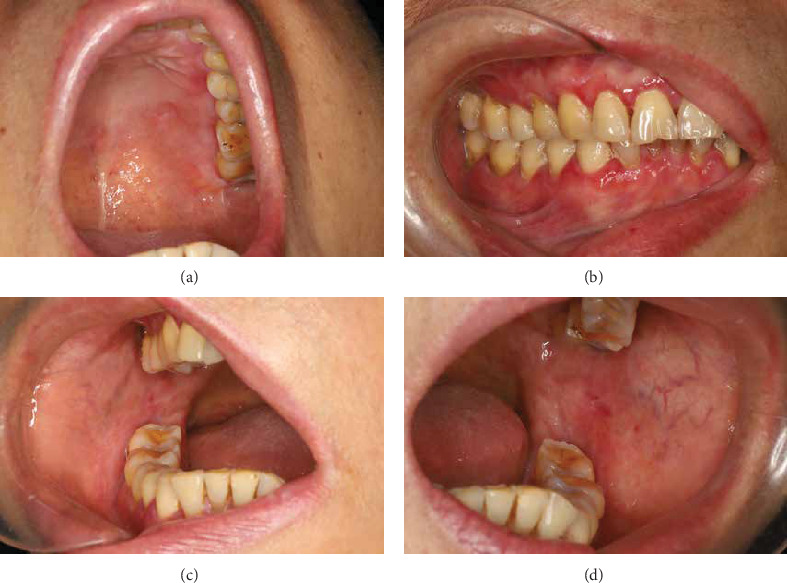
Clinical images of left palatal mucosa (a), right maxillary and mandibular gingiva (b), right buccal mucosa (c), and left buccal mucosa (d), at 6 months.

**Figure 5:**
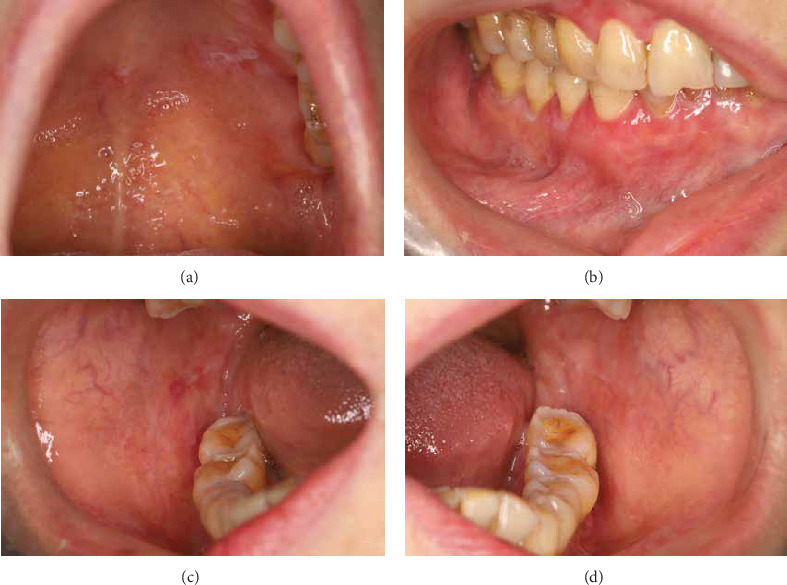
Clinical images of left palatal mucosa (a), right maxillary and mandibular gingiva (b), right buccal mucosa (c), and left buccal mucosa (d), at 14 months.

After 4 weeks, the patient experienced significant symptomatic improvement. Investigator Global Assessment [26] was reduced from 3 to 1 after 4 weeks of treatment (Table 1). The reticulation hyperemia and ulceration score for oral LP [27] reduced from 4.26 to 1.3. The patient reported headache and gastrointestinal symptoms at 2 weeks after treatment, which improved within 4 weeks. Moreover, the patient reported improved sleep and well tolerance with tooth brushing and vulvovaginal examination. At the 8-week follow-up (Figure 2), periodontal scaling and root planing were performed, considering the patient's disease was amenable to this treatment (Figure 3). The dosage of apremilast was reduced from 60 to 30 mg per day. At the 6-month follow-up, the patient's condition was stable (Figure 4), and tests including complete blood count, HCV RNA load, thyroid function, liver function, renal function and coagulation profile showed no significant abnormalities. At the 14-month follow-up after the initiation of treatment, the patient's condition remained stable (Figure 5) and she reported satisfaction with the improvement in her quality of life. The patient's therapeutic course is summarized in detail in Table 2.

## 3. Discussion

There are currently no FDA-approved therapies for LP and off-label therapeutic algorithms have been proposed [1]. Given its refractory nature, systemic therapies are necessary for managing generalized LP which can affect multiple mucosal and skin sites simultaneously or successively. Previous case reports have reported that oral administration of apremilast effectively improves the condition of LP patients [16, 17, 28]. Our patient exhibited long-standing LP that was resistant to standard treatments, which is similar to the refractory condition reported in previous cases. However, improvement with apremilast in patients with LP involving the gingiva, vagina, and vulva has not been reported. Our patient's clinical symptoms were aligned with the “vulvovaginal gingival syndrome” [29, 30]. Currently, there are no consensus guidelines for the management of plurimucosal LP, including challenging syndromes such as vulvovaginal–gingival syndrome. The absence of standardized therapeutic approaches highlights the need for innovative management strategies to address these difficult cases. The sequential therapy regimen described in this report offers an example of a multidisciplinary and stepwise approach that warrants further investigation to determine its broader applicability and to inform future guideline development.

Apremilast increases intracellular cAMP which is critical for the production of several proinflammatory cytokines [31]. Previously, we demonstrated that IFN-*γ* aggravated the LP via priming basal keratinocytes [32]. Given the critical role of IFN-*γ* and T helper 1 cells in numerous cutaneous and oral mucosal inflammatory conditions, we hypothesized that apremilast should also be effective for LP. Moreover, we previously demonstrated that TGP enhanced the efficacy and lowered the side effects in a randomized clinical trial for psoriasis [33]. Mechanism research indicated that TGP inhibits TNF-*α* and IL-6 production by suppressing the NF-*κ*B signaling pathway in oral LP [22]. TGP has immunomodulatory effects; however, its therapeutic efficacy requires a prolonged duration to manifest.

Periodontal disease may affect the treatment efficacy of oral LP [34]. In patients with oral LP lesions, particularly those with the erosive phenotype, maintaining effective oral hygiene is often compromised due to pain and discomfort, leading to dental plaque accumulation, which exacerbates inflammation [9, 10, 12]. The novel aspect of our approach was the strategic timing of the intervention. In this case, systemic therapy with apremilast and TGP was first initiated to control the acute inflammatory pain and lesions. And when patients are able to tolerate it, basic periodontal treatment can effectively reduce these associated risk factors. In our case, we performed hygiene measures at the 8-week follow-up when this patient's symptoms had significantly improved. As part of a sequential treatment approach, the patient in this case underwent both supragingival and subgingival scaling after improvement of the oral lesions. This stepwise approach addresses both systemic and local contributing elements of the disease.

This report describes a single patient, so the findings must be interpreted with caution. No biopsy was obtained because the clinical appearance was typical and the patient declined invasive procedures; consequently, histopathological confirmation is lacking. Furthermore, whether the sequential apremilast-TGP approach can be generalized to other patients with plurimucosal LP is unknown. Prospective trials with larger sample sizes, objective histological endpoints, and standardized adverse event monitoring are needed to assess its broader applicability. Additionally, comparative trials evaluating this protocol against standard immunosuppressants will provide insights into its efficacy, safety, and optimal treatment duration.

In summary, the sequential therapy regimen comprising apremilast, TGP, and periodontal treatment may have achieved a synergistic effect on the immunomodulatory and anti-inflammatory functions in a patient with refractory LP. This integrated approach appeared to address the underlying inflammatory processes while also improving the local oral environment, which might contribute to the overall stability of the condition. However, as this is a single case report, these findings cannot be generalized, and no definitive conclusions can be drawn. This experience suggests a potential therapeutic strategy worthy of further investigation in larger, controlled studies to validate its efficacy and mechanism.

## Figures and Tables

**Table 1:** Disease severity and pain scores before and after treatment in this patient.

	**REU**	**VAS**	**IGA**
Before treatment	4.26	8	3
1 month	1.30	2	1
2 months	1.18	1	1
6 months	1.06	1	1
14 months	1.14	0	1

*Note:* Visual analog scale (VAS) for pain: score 0–10; investigator global assessment (IGA): score 0–4.

Abbreviations: IGA, Investigator Global Assessment; REU, reticulation–erythema–ulceration; VAS, visual analog scale.

**Table 2:** Timeline of clinical response and treatment milestones in this patient.

**Date**	**Key clinical findings**	**Treatment initiated/modified**	**Milestones and notes**
2019–2023	White striae, erythema, erosion, vulvovaginal lesions	Hydroxychloroquine sulfate + submucosal glucocorticoid injection + topical triamcinolone acetonide + TGP + topical tacrolimus ointment	Disease recurrent; side effects related to immunosuppressants
2024/1/19	White striae, erythema, erosion, vulvovaginal lesions, nail lesions	TGP + apremilast + tacrolimus ointment	First use of apremilast
2024/2/21	White striae, erythema, vulvovaginal lesions, nail lesions	TGP + apremilast + tacrolimus ointment	Erosion healed, erythema area reduced
2024/3/27	Slightly white striae; slightly erythema	Apremilast + periodontal scaling	Disease stable, combined with periodontal therapy
2024/7/16	Slightly white striae; slightly erythema	Apremilast dose reduced (30 mg, once daily)	Lesions remained stable
2025/3/19	Slightly white striae; slightly erythema	Apremilast dose reduced (30 mg, every other day)	Lesions remained stable
2025/9/22	—	Apremilast (30 mg, every other day)	Telephone follow-up: lesions continued stability

## Data Availability

The data that support the findings of this study are available from the corresponding author upon reasonable request.

## Nomenclature

cAMP: cyclic adenosine monophosphate
FDA: Food and Drug Administration
IFN-*γ*: interferon gamma
IL: interleukin
LP: lichen planus
PDE-4: phosphodiesterase-4
TGP: total glucosides of paeony
TNF-*α*: tumor necrosis factor-alpha

## References

[1] Louisy A., Humbert E., Samimi M. (2024). Oral Lichen Planus: An Update on Diagnosis and Management. *American Journal of Clinical Dermatology*.

[2] Tekin B., Xie F., Lehman J. S. (2024). Lichen Planus: What Is New in Diagnosis and Treatment?. *American Journal of Clinical Dermatology*.

[3] Rosset F., Sciamarrelli N., Mastorino L. (2025). Monoclonal Antibodies and Small-Molecule Therapies for Lichen Planus: Targeted Immunomodulation and Emerging Evidence. *Antibodies*.

[4] Manchanda Y., Rathi S. K., Joshi A., Das S. (2024). Oral Lichen Planus: An Updated Review of Etiopathogenesis, Clinical Presentation, and Management. *Indian Dermatology Online Journal*.

[5] Vičić M., Hlača N., Kaštelan M., Brajac I., Sotošek V., Prpić Massari L. (2023). Comprehensive Insight Into Lichen Planus Immunopathogenesis. *International Journal of Molecular Sciences*.

[6] Deng X., Wang Y., Jiang L., Li J., Chen Q. (2023). Updates on Immunological Mechanistic Insights and Targeting of the Oral Lichen Planus Microenvironment. *Frontiers in Immunology*.

[7] Garcia-Pola M. (2023). Bidirectional Association Between Lichen Planus and Hepatitis C-an Update Systematic Review and Meta-Analysis. *Journal of Clinical Medicine*.

[8] Brzdęk M., Gałuszka-Garnuszek J., Dobrowolska K. (2025). Oral Manifestations in Patients With Chronic Hepatitis C. *BMC Oral Health*.

[9] Schellinck A. E., Rees T. D., Plemons J. M., Kessler H. P., Rivera-Hidalgo F., Solomon E. S. (2009). A Comparison of the Periodontal Status in Patients With Mucous Membrane Pemphigoid: A 5-Year Follow-up. *Journal of Periodontology*.

[10] Ramon‐Fluixa C., Bagán‐Sebastián J. V., Milián‐Masanet M. A., Scully C. (1999). Periodontal Status in Patients With Oral Lichen Planus: A Study of 90 Cases. *Oral Diseases*.

[11] Sanadi R. M., Khandekar P. D., Chaudhari S. R., Javali M. A., Gurav N. U. (2023). Association of Periodontal Disease With Oral Lichen Planus: A Systematic Review and Meta Analysis. *Journal of Oral and Maxillofacial Pathology*.

[12] Rai N. P., Kumar P., Mustafa S. M. (2016). Relation Between Periodontal Status and Pre-Cancerous Condition (Oral Lichen Planus): A Pilot Study. *Advances in Clinical and Experimental Medicine*.

[13] Mehta H., Sharma A., Dogra S. (2022). Evolving Utility of Apremilast in Dermatological Disorders for Off-Label Indications. *Clinical and Experimental Dermatology*.

[14] Perschy L., Anzengruber F., Rappersberger K. (2022). Apremilast bei Oralem Lichen Planus - eine Multizentrische, Retrospektive Studie. *Journal der Deutschen Dermatologischen Gesellschaft*.

[15] Viswanath V., Joshi P., Dhakne M., Dhoot D., Mahadkar N., Barkate H. (2022). Evaluation of the Efficacy and Safety of Apremilast in the Management of Lichen Planus. *Clinical, Cosmetic and Investigational Dermatology*.

[16] Sil S., Shome S., Saha N., Ghosh S. (2023). Apremilast in Oral Lichen Planus: Report of Two Cases and Review of Literature. *Indian Journal of Dermatology*.

[17] AbuHilal M., Walsh S., Shear N. (2016). Treatment of Recalcitrant Erosive Oral Lichen Planus and Desquamative Gingivitis With Oral Apremilast. *Journal of Dermatological Case Reports*.

[18] Paul J., Foss C. E., Hirano S. A., Cunningham T. D., Pariser D. M. (2013). An Open-Label Pilot Study of Apremilast for the Treatment of Moderate to Severe Lichen Planus: A Case Series. *Journal of the American Academy of Dermatology*.

[19] Ren L., Zhao K., Wang B., Xiao S., Liu J., Tu C. (2025). Safety Assessment of Apremilast: Real-World Adverse Event Analysis From the FAERS Database. *Archives of Dermatological Research*.

[20] Tello E. D., Suárez J. A., Catalán E. B. (2021). Multidisciplinary Management of the Adverse Effects of Apremilast. *Actas Dermo-Sifiliográficas (English Edition)*.

[21] Jiang H., Li J., Wang L. (2020). Total Glucosides of Paeony: A Review of Its Phytochemistry, Role in Autoimmune Diseases, and Mechanisms of Action. *Journal of Ethnopharmacology*.

[22] Wang Y., Zhang H., du G. (2016). Total Glucosides of Paeony (TGP) Inhibits the Production of Inflammatory Cytokines in Oral Lichen Planus by Suppressing the NF-*κ*B Signaling Pathway. *International Immunopharmacology*.

[23] Zhao Z., Han Y., Zhang Z. (2018). Total Glucosides of Paeony Improves the Immunomodulatory Capacity of MSCs Partially via the miR-124/STAT3 Pathway in Oral Lichen Planus. *Biomedicine & Pharmacotherapy*.

[24] Zhao M., Peng N., Zhou Y. (2025). The Immunoregulatory Effects of Total Glucosides of peony in Autoimmune Diseases. *Journal of Leukocyte Biology*.

[25] Zhou L., Cao T., Wang Y. (2016). Clinical Observation on the Treatment of Oral Lichen Planus With Total Glucosides of Paeony Capsule Combined With Corticosteroids. *International Immunopharmacology*.

[26] Brennan M. T., Riordain R. N., Long-Simpson L., Bissonnette C., Lizano M., Madsen L. S. (2023). Oral Lichen Planus Clinician Reported Outcome Measure: Development, Content Validity, and Further Development. *Oral Diseases*.

[27] Wu Y., Xu H., Wang Y. (2023). An Improved Scoring System for Monitoring Oral Lichen Planus: A Preliminary Clinical Study. *Oral Diseases*.

[28] Kim-Lim P., Thomas C. (2023). Crushed Apremilast for the Treatment of Oral Lichen Planus. *JAAD Case Reports*.

[29] Bermejo A., Bermejo M. D., Román P., Botella R., Bagán J. V. (1990). Lichen Planus With Simultaneous Involvement of the Oral Cavity and Genitalia. *Oral Surgery, Oral Medicine, and Oral Pathology*.

[30] Shao Y., Sun K., Yang M., Chang J. (2024). Vulvar-Vaginal-Gingival-Otic Syndrome. *Experimental Dermatology*.

[31] Oehrl S., Prakash H., Ebling A. (2017). The Phosphodiesterase 4 Inhibitor Apremilast Inhibits Th1 but Promotes Th17 Responses Induced by 6-Sulfo LacNAc (slan) Dendritic Cells. *Journal of Dermatological Science*.

[32] Shao S., Tsoi L. C., Sarkar M. K. (2019). IFN-*γ* Enhances Cell-Mediated Cytotoxicity Against Keratinocytes Via JAK2/STAT1 in Lichen Planus. *Science Translational Medicine*.

[33] Yu C., Fan X., Li Z., Liu X., Wang G. (2017). Efficacy and Safety of Total Glucosides of Paeony Combined With Acitretin in the Treatment of Moderate-to-Severe Plaque Psoriasis: A Double-Blind, Randomised, Placebo-Controlled Trial. *European Journal of Dermatology*.

[34] Wu T., Bai Y., Jing Y., Chen F. (2024). What Can we Learn From Treatments of Oral Lichen Planus?. *Frontiers in Cellular and Infection Microbiology*.

